# Effectiveness analysis of herbal medicine for gastroesophageal reflux disease: A retrospective study

**DOI:** 10.1097/MD.0000000000037295

**Published:** 2024-02-23

**Authors:** Hesol Lee, Changsub Yang, Ojin Kwon, Ki-Byoung Kim, Wongu Lee, Sungha Kim

**Affiliations:** a Clinical Research Coordinating Team, Korea Institute of Oriental Medicine, Daejeon, Republic of Korea; b KM Science Research Division, Korea Institute of Oriental Medicine, Daejeon, Republic of Korea; c The Association of Korean Medicine, Daejeon Metropolitan, Republic of Korea.

**Keywords:** case series, gastroesophageal reflux disease, herbal medicine, Korean medicine clinics

## Abstract

This study aimed to identify the clinical features of gastroesophageal reflux disease (GERD) in primary clinics and the effectiveness and safety of herbal medicine (HM). Thirty-five patients with gastroesophageal-reflux symptom who visited the 16 Korean medicine (KM) primary clinics from June 2022 to October 2022 were included in the study. We retrospectively analyzed the charts of 35 patients and collected clinical characteristics, HM, and outcome variables such as the numerical rating scale, gastroesophageal reflux disease questionnaire, frequency scale for symptoms of gastroesophageal reflux disease, Euro-Quality Of Life-5 Dimension, and adverse events. Of the 35 patients, 12 (34.3%) were men, and the average age of all patients was 47.0 ± 14.3 years. HM was prescribed for all 35 patients; Pinelliae Tuber (n = 31, 88.57%), Zingiberis Rhizoma Recens (n = 30, 85.71%), and Poria Sclerotium (n = 28, 80%) were the most prescribed herbs. All scores including numerical rating scale, frequency scale for symptoms of gastroesophageal reflux disease, gastroesophageal reflux disease questionnaire, and Euro-Quality Of Life-5 Dimension after 30 and 60 days from baselines showed significant improvement, and there were only a few adverse events. This study supports the effectiveness and safety of HM in reducing GERD symptoms in primary Korean medicine clinics. The most frequently used herbs may play significant roles in GERD symptom management.

## 1. Introduction

Gastroesophageal reflux disease (GERD) is a condition where the contents of the stomach reflux into the esophagus, causing discomfort or complications.^[1]^ GERD is a common disease, ranking seventh in frequency among outpatient visits according to 2021 data from the Health Insurance Review and Assessment Service, with approximately 4.9 million patients, and is one of the main reasons for hospital visits, with a 10.0% increase in medical expenses compared to 2020 in South Korea.^[2]^ In particular, according to the 2018 National Health Insurance Service statistics on the most common illnesses by International Statistical Classification of Diseases and Related Health Problems, there were nearly 20,000 outpatient visits solely for the K21 code, GERD.^[3]^

Although various treatment options are available for GERD, there are limitations and disadvantages associated with existing approaches. Commonly used treatments such as proton pump inhibitors provide symptom relief but may not address the underlying causes of GERD or prevent disease progression.^[4]^ Moreover, long-term proton pump inhibitor use has been associated with adverse effects such as infections, impaired nutrient absorption, dementia, kidney disease, and hypergastrinemia-related side effects.^[5]^ Various drugs based on various pathophysiological mechanisms, such as the use of drugs that enhance mucosal recovery, have been proposed, but the evidence for this is still limited.^[6]^ This highlights the need for alternative and complementary treatment approaches that can provide sustained relief with fewer side effects.

In the context of treating GERD, herbal medicine (HM) offers a potential alternative approach that deserves attention. A survey conducted in 2020 by the Korean Ministry of Health and Welfare reported that approximately 69% of Koreans have sought Korean medicine (KM) treatment, representative of HM, with 10.3% specifically seeking it for digestive disorders.^[7]^ A retrospective study published in 2021 analyzed the medical records of 1151 patients with GERD who received KM including HM at hospital as an intervention for GERD and found that most patients visiting KM hospitals were females in their sixties with chronic and refractory reflux esophagitis.^[8]^ Several studies have explored the effectiveness of HM interventions for GERD. A case series of patients with GERD treated with *Ljintang-Gamibang* and acupuncture showed that HM with acupuncture significantly reduced the patients’ symptoms and improved their Quality Of Life (QOL).^[9]^ Another study also showed HM was effective for refractory GERD.^[10]^ These findings, supported by both clinical practice and research, highlight the potential of HM as a valuable addition to the treatment options available for patients with GERD. However, none of the studies investigated diverse HM usage in real-world practice for patients with GERD in multi-primary KM clinics.

KM doctors primarily handle the treatment of disease in primary healthcare institutions. As of 2022, there are 513 KM hospitals and 14,558 KM clinics in operation, with 97% of KM care facilities being clinics. In terms of medical expenses, KM hospitals account for 0.3438 trillion Won and KM clinics account for 1.8472 trillion Won, with over 84% of treatments being provided at the clinic level.^[11]^ This highlights the need for research focused on clinical practitioners in primary KM clinics. Jung et al^[12]^ raised concerns about the problem of unreflected characteristics of actual clinical practice in clinical research in KM, which has various types of treatment processes that are customized to individual patients rather than diseases, and include treatment processes that involve the relationship between doctors and patients. They suggested involving primary care physicians, who represent the majority of KM practitioners, in clinical research, rather than limiting the subject of traditional KM clinical research to university hospitals.^[12]^ Research based on retrospective analysis of clinical experience and medical records in the field is necessary to identify treatment effects and side effects, and to propose new treatment approaches.

This study is a retrospective analysis of the medical records of 35 patients who received HM for GERD at KM clinics. We aimed to determine the clinical effectiveness and safety of HM for GERD through chart reviews. The study aims to confirm the maintenance of symptoms in patients with GERD 1 month after treatment completion. These results will be utilized to analyze the effects of HM on GERD symptom intensity, improvement of specific GERD symptoms, and QOL. Additionally, this study shows the diverse HM usage for patients with GERD. This would provide the principal herbs for the management of GERD symptoms.

## 2. Methods

### 2.1. Study participants and methods

This was a multicenter retrospective chart review that collected medical records of patients who received outpatient HM at 18 local primary KM clinics from June 2022 to October 2022 and met the following criteria:

#### 2.1.1. Selection criteria.

1. Patients who had record patterns using Pattern identification of GERD tool, numerical rating scale (NRS) for GERD symptoms, frequency scale for symptoms of gastroesophageal reflux disease (FSSG), gastroesophageal reflux disease questionnaire (GerdQ), and Euro-Quality Of Life-5 Dimension 5-Level version (EQ-5D-5L)
2. Patients with GerdQ, FSSG scores of 8 or higher, and NRS scores of 4 or higher
3. Patients with a medication adherence rate of 80% or higher

#### 2.1.2. Exclusion criteria.

1. Patients who have received other interventions during the treatment period
2. Patients with missing records that make evaluation difficult

This study identified 35 patients who met the above criteria and agreed to allow access to their medical records for research purposes and signed a consent form for the third-party provision and use of personal information for research purposes during their visit to the selected 18 local KM clinics. This study was conducted with the approval of the institutional review board (I-2303/003-001).

### 2.2. Research method

Thirty-five patients who received HM for 30 days and returned for follow-up observation 30 days after HM completion met the inclusion criteria. Their medical charts were retrospectively analyzed, and the following items were collected for each patient if recorded:

(1) General characteristics of the patient: date of birth, sex, weight, height, and lifestyle habits (caffeine intake, smoking, alcohol consumption)
(2) Medical history related to GERD and other diseases: treatment content, treatment period, and related medication history
(3) Pattern identification of GERD tool^[13]^: A symptom questionnaire consisting of 32 self-reporting items for patients to accurately reflect their own symptoms, and 8 objective items for physicians to use in the diagnosis and treatment of GERD. The scores of the questionnaire items were calculated, and the highest score among the 4 types of GERD patterns (Liver Qi Invading the Stomach, Spleen-stomach weakness pattern, Spleen-Stomach Dampness-Heat, Stomach yin deficiency pattern) was selected as the patient’s pattern type.
(4) Prescribed HM, medication adherence, and treatment satisfaction
(5) Number of visits and treatment period
(6) Pre- and posttreatment comparison indicators:
  (1) NRS^[14]^ for GERD symptoms: A 10-point scale where 0 indicates “no pain or discomfort” and 10 indicates “pain or discomfort as severe as possible.”
  (2) FSSG^[15]^: A scale used to measure the frequency of GERD symptoms. It can be used to determine the severity of GERD.^[16]^ Twelve questions and most often answered “yes” were selected, assigned scores (never = 0; occasionally = 1; sometimes = 2; often = 3; and always = 4). As total score set over 8 points, this questionnaire showed a sensitivity of 62%, a specificity of 59%, and an accuracy of 60%.^[15]^
  (3) GerdQ^[17]^: The Korean version of GerdQ consists of 6 items and can be easily administered to suspected GERD patients for initial diagnosis or posttreatment follow-up.^[8]^ The questionnaire comprises of 4 positive predictors of GERD (heartburn, regurgitation, sleep disturbance, and use of over-the-counter medication in addition to that prescribed) and 2 negative predictors of GERD (epigastric pain and nausea). Items were graded; scores ranging from 0 to 3 were applied for the positive predictors and from 3 to 0 for the negative predictors and calculated as the sum of all scores 0 to 18. The score 8 was found to have the highest sensitivity (64.9%; 95% CI, 56.2–73.7) and specificity (71.4%; 95% CI, 56.5–86.4) for the diagnosis of GERD.^[18]^
  (4) EQ-5D-5L: EQ-5D-5L is a standardized instrument for measuring health-related QOL. It is a self-administered questionnaire that consists of 2 parts. The first part assesses 5 dimensions of health: mobility, self-care, usual activities, pain/discomfort, and anxiety/depression. Each dimension has 5 levels of response, ranging from no problems to extreme problems. The second part of the questionnaire is a visual analogue scale (VAS) that measures overall health status on a scale from 0 (worst imaginable health state) to 100 (best imaginable health state).
(7) Adverse reactions

### 2.3. Statistical analysis

#### 2.3.1. General principles of statistical analysis.

For continuous data, such as demographic and sociological information of the patients included in this study, descriptive statistics such as mean, standard deviation, 95% confidence interval, minimum, and maximum values are presented. For categorical data, frequencies and percentages are presented. Analysis of HM prescriptions involves identifying frequently used herbs based on the number of prescriptions and the weight (g) of each herb. In the case of combination prescriptions, the primary prescription is used as a reference, and subgroup analysis is performed for cases where the prescription changes during the course of treatment.

#### 2.3.2. Statistical analysis method for evaluation variables.

**Figure 1.**
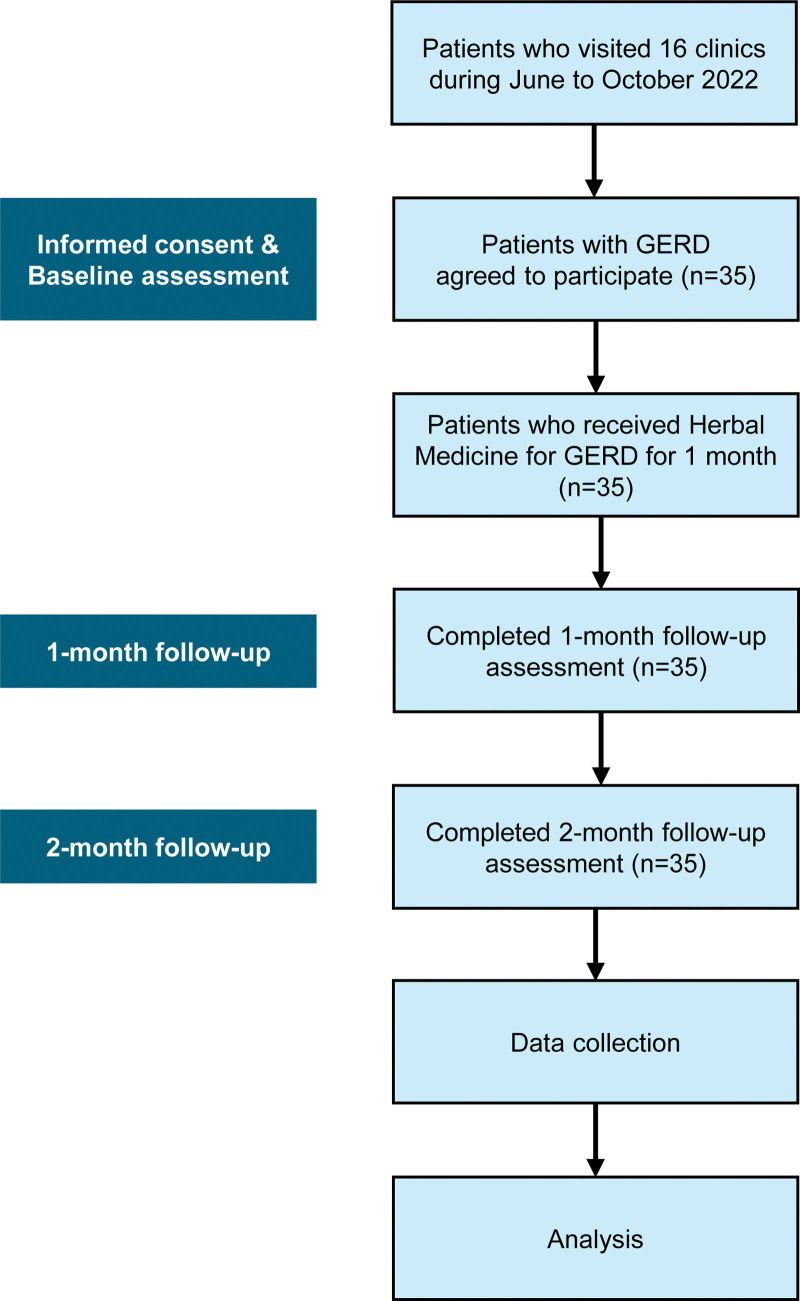
Flowchart. Participants received Korean medicine treatment by experienced Korean Medicine doctors. Korean medicine treatment was only herbal medicine, and all clinics already had the data of outcome assessments. GERD = gastroesophageal reflux disease.

Major treatment effect evaluation variables were GerdQ, FSSG, NRS for GERD Symptoms, GERD Assessment Tool, and EQ-5D. To compare the difference before and after treatment for all subjects, a paired *t* test or Wilcoxon signed-rank test is performed depending on the normality of the data. A *P*-value of <0.05 is considered statistically significant. The analysis involves comparing the difference scores of GerdQ and FSSG and the change between each score, as well as analyzing the correlation between NRS and EQ-5D. Comparison analysis of caffeine intake, smoking, and alcohol consumption was performed for subgroup comparison analysis. If necessary, an independent *t* test or Wilcoxon rank sum test was performed to compare the difference before and after treatment depending on the normality of the data. The safety evaluation summarizes the types and frequencies of collected adverse reactions. Figure 1 shows the flowchart of the study performed.

## 3. Results

### 3.1. Baseline characteristics

Thirty-five patients diagnosed with GERD were included from the 16 KM primary clinics. The patients visited the clinics every 4 weeks for their HM prescriptions. Among the total of 35 patients, 65.7% were women and 34.3% were men. The mean age of patients with GERD and the duration of GERD were 47.0 ± 14.3 and 5.1 ± 5.4 years, respectively. The average height, weight, and body mass index of the participants were 164.9 ± 7.6 cm, 62.7 ± 13.5 kg, and 22.8 ± 3.5 kg/m^2^, respectively.

Patients with GerdQ and FSSG scores of 8 or higher and NRS scores of 4 or higher were included in the study. The average scores for NRS was 6.8 ± 1.2, for GerdQ was 10.1 ± 1.6, and for FSSG was 22.2 ± 6.5.

We summarized the general characteristics of 35 patients including patterns of caffeine and alcohol intake, smoking, treatment history, and pattern identification of GERD according to the stages in Table 1.

**Table 1:** Characteristics of patients with GERD (n = 35).

Variables		Number of patients (%) or mean ± SD
Sex	Male	12 (34.3%)
	Female	23 (65.7%)
Age		47.0 ± 14.3
Disease duration (yr)		5.1 ± 5.4
Height		164.9 ± 7.6
Weight		62.7 ± 13.5
BMI		22.8 ± 3.5
Caffeine intake	Y	26 (74.3%)
	N	9 (25.7%)
	(Glass/d)	1.9 ± 1.3
Alcohol drinking	Y	14 (40.0%)
	N	21 (60.0%)
		7.1 ± 8.0
Smoke	Y	1 (2.9%)
	N	34 (97.1%)
Medical history		
	Drug	9 (25.7%)
	Health functional food	2 (5.7%)
	Acupuncture	7 (20.0%)
	Moxibustion	7 (20.0%)
	Cupping	5 (14.3%)
	Herbal medicine	5 (14.3%)
	Health food	7 (20.0%)
Pattern		
	Liver Qi Invading the Stomach	19 (54.3%)
	Spleen-stomach weakness pattern/syndrome	5 (14.3%)
	Spleen-Stomach Dampness-Heat	8 (22.9%)
	Stomach yin deficiency pattern/syndrome	3 (8.6%)

BMI = body mass index, N = No, SD = standard deviation, Y = Yes.

### 3.2. HM used for patients with GERD

In this study, participants with a medication adherence rate of 80% or higher were included in the effectiveness evaluation analysis. As a result, all participants met the adherence criterion, with an average adherence rate of 95.9%.

Table 2 provides HM applied for patients with GERD in this study, includes a detailed description of HM provided. Thirty-two cases (91.43%) used formulae in the standard or added form. As for the formula used, of the 35 total patients, 8 patients were prescribed with *Gaewool-Whadam-Jian*, 7 patients with *Jeungmiyijin-tang*, 5 patients with *Yukul-tang Gamibang*, and 12 patients with *Hyangsayukgunja-tang*. Three cases were excluded from Table 2 because 1 case used *Taeeumjowi-tang* which had minimal inclusion of herbal ingredients commonly used for GERD in the constitutional prescription and 2 cases involved a combination of various medications.

**Table 2:** Herbal medicine used for patients with GERD.

Name of herbal medicine	Variables	Number of participants (%)
Herbal medicine^a^		
*Gaewool-Whadam-Jian*	Cyperi Rhizoma 9.375 g, Pinelliae Tuber, Zingiberis Rhizoma Recens, Poria Sclerotium, Citri Unshius Pericarpium, Coptidis Rhizoma, Massa Medicata Fermentata, Atractylodis Rhizoma, Ponciri Fructus Immaturus, Raphani Semen, Forsythiae Fructus, Magnoliae Cortex 3.75 g, Aucklandiae Radix 2.625 g, Scutellariae Radix 1.875 g	8 (22.86)
*Jeungmiyijin-tang*	Pinelliae Tuber, Zingiberis Rhizoma Recens, Poria Sclerotium, Citri Unshius Pericarpium, Cyperi Rhizoma, Coptidis Rhizoma, Gardeniae Fructus, Ponciri Fructus Immaturus 4 g, Atractylodis Rhizoma, Cnidii Rhizoma 3.2 g, Paeoniae Radix 2.8 g, Massa Medicata Fermentata 2 g, Glycyrrhizae Radix et Rhizoma 1.2 g	7 (20)
*Yukul-tang Gamibang*	Cyperi Rhizoma 8 g, Atractylodis Rhizoma, Cnidii Rhizoma 6 g, Pinelliae Tuber, Zingiberis Rhizoma Recens, Citri Unshius Pericarpium, Massa Medicata Fermentata, Hordei Fructus Germinatus, Crataegi Fructus 4 g, Poria Sclerotium, Gardeniae Fructus 2.8 g, Glycyrrhizae Radix et Rhizoma, Amomi Fructus 2 g	5 (14.29)
*Hyangsayukgunja-tang*	Pinelliae Tuber, Poria Sclerotium, Ginseng Radix, Atractylodis Rhizoma Alba 8 g, Citri Unshius Pericarpium, Zingiberis Rhizoma Recens, Zizyphi Fructus, Glycyrrhizae Radix et Rhizoma 4 g, Aucklandiae Radix, Amomi Fructus 3.2 g	12 (34.29)
Herbs most added^b^		
	Pinelliae Tuber	31 (88.57)
	Zingiberis Rhizoma Recens	30 (85.71)
	Poria Sclerotium	28 (80)
	Citri Unshius Pericarpium	26 (74.29)
	Cyperi Rhizoma	23 (65.71)
	Coptidis Rhizoma	23 (65.71)
	Massa Medicata Fermentata	21 (60)
	Atractylodis Rhizoma	21 (60)
	Glycyrrhizae Radix et Rhizoma	20 (57.14)
	Magnoliae Cortex	20 (57.14)

a Herbs presented at variables are the standard formula. All formulae were water extract decoctions and used in the standard or herbs added form. *Yukul-tang Gamibang* is for dietetic stagnation (食鬱) pattern.

b Herbs frequently added to the standard formula are presented below the frequency of the formula used. Herbs added to the standard formula >20 times are shown in this table. Other herbs were added to the standard formula <20 times.

**Figure 2.**
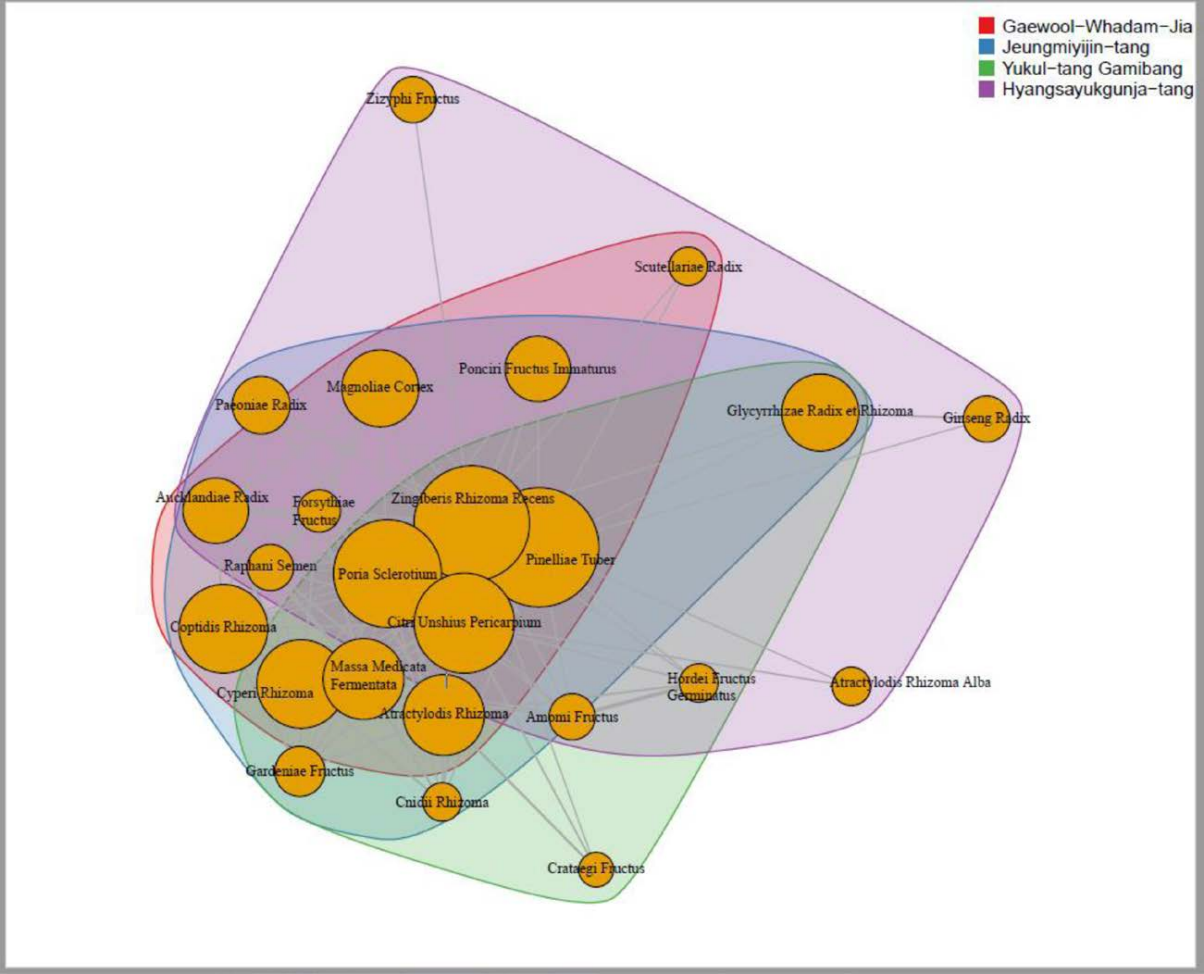
Network visualization of frequently used herbs in the herbal decoction for patients with GERD. The size of the nodes represents the frequency of the herb used in the decoction, while the distance between nodes represents the association between herbs. Grouping was done with community detection, and as a result, the large orange nodes in the core are composed of herbs frequently used for GERD, regardless of the Korean medicine type. The nodes inside the purple color were mainly composed of qi-tonifying medicinal herbs, whereas the nodes within the green color seem to be digestion-promoting medicine herbs. GERD = gastroesophageal reflux disease.

The commonly added herbs and the frequency of the formula used are presented in Table 2. Figure 2 presents the network visualization of the frequently used herbs in the herbal decoction formulae and their grouping by community detection. The frequency of each herb in decoction is represented by the size of node, and the association between herbs is represented by the distance of nodes. Table 2 shows that the herbs most frequently added (>50%) to the standard herbal decoction, regardless of the formula, were Pinelliae Tuber, Zingiberis Rhizoma Recens, Poria Sclerotium, Citri Unshius Pericarpium, Cyperi Rhizoma, Coptidis Rhizoma, Massa Medicata Fermentata, Atractylodis Rhizoma, Glycyrrhizae Radix et Rhizoma, and Magnoliae Cortex. As shown in Figure 2, Pinelliae Tuber, Zingiberis Rhizoma Recens, Poria Sclerotium, Citri Unshius Pericarpium, and Cyperi Rhizoma are located in the core part (center node with 4 overlapping parts) of the network, which were the herbs most frequently added to the formulae for patients with GERD.

We gathered pattern identification of GERD and investigated its correlation with the formula. However, we were unable to confirm the correlation with a sample size of 35 (correlation coefficient: 0.09). On the other hand, the study demonstrated that 54.3% of the observed cases exhibited characteristics indicative of Liver Qi Invading the Stomach, while 22.9% of the cases displayed features consistent with Spleen-Stomach Dampness-Heat. Notably, these 2 identified patterns accounted for a cumulative proportion of 77.2% among the total cases examined. Liver Qi Invading the Stomach symptoms are belching, throat pain, a bitter taste, being easily upset and irritable, and dry mouth; while Spleen-Stomach Dampness-Heat symptoms are rapid pulse, greasy yellow coating tongue, tongue redness, yellow urine, burning epigastric pain.^[13]^

### 3.3. GERD scores: NRS, GerdQ, FSSG, EQ-5D

Decreasing trends of NRS, GerdQ, and FSSG scores over time means improvement of GERD, and increase of EQ-5D means improvement of QOL. The mean NRS score was 6.8 ± 1.2 at the baseline and decreased to 3.4 ± 2.0 and 2.3 ± 1.4 after 30 and 60 days, respectively, which were statistically significant (both *P* < .0001; Table 3). The mean GerdQ score at baseline and after 30 and 60 days of treatment was, 10.1 ± 1.6, 5.9 ± 3.4, and 4.6 ± 2.7, respectively. These differences were statistically significant with a *P*-value of *P* < .0001 (Table 3).

**Table 3:** Changes in the NRS, GerdQ, FSSG, and EQ-5D scores.

	Baseline (n = 35)	After 30 d (n = 35)	After 60 d (n = 35)	*P*-value	
				30 d^a^	60 d^b^
NRS	6.8 ± 1.2	3.4 ± 2.0	2.3 ± 1.4	<.0001*	<.0001*
GerdQ	10.1 ± 1.6	5.9 ± 3.4	4.6 ± 2.7	<.0001*	<.0001*
FSSG	22.2 ± 6.5	11.4 ± 5.3	8.4 ± 5.5	<.0001*	<.0001*
EQ-5D-5L	0.841 ± 0.092	0.902 ± 0.092	0.934 ± 0.089	<.0001*	<.0001*
EQ-VAS	64.3 ± 18.9	73.9 ± 18.6	79.5 ± 15.1	<.0001*	<.0001*

Values are expressed as mean ± standard deviation. *P*-values are calculated by Student paired *t* test or Wilcoxon signed rank test, depending on the normality.

EQ-5D-5L = Euro-Quality Of Life - 5 Dimension – 5 Level, EQ-VAS = Euro-Quality Of Life visual analogue scale, FSSG = Frequency Scale for the Symptoms of GERD, GerdQ = gastroesophageal reflux disease questionnaire, NRS = numerical rating scale.

a Comparing measurement before the intervention (baseline) and after 30 d.

b Comparing measurement before the intervention (baseline) and after 60 d.

The FSSG score at baseline and after 30 and 60 days of treatment was 22.2 ± 6.5, 11.4 ± 5.3, and 8.4 ± 5.5, respectively. These differences were statistically significant with a *P*-value of *P* < .0001 for both (Table 3). The mean EQ-5D-5L score was 0.841 ± 0.092 at baseline and increased to 0.902 ± 0.092 and 0.934 ± 0.089 after 30 and 60 days, respectively. These differences were statistically significant (both *P* < .0001; Table 3). EQ-VAS score was 64.3 ± 18.9 at baseline and increased to 73.9 ± 18.6 and 79.5 ± 15.1, after 30 and 60 days, respectively. These differences were statistically significant (both *P* < .0001; Table 3).

### 3.4. Subgroup comparison analysis

Subgroup comparison analysis was performed to evaluate the treatment effectiveness based on lifestyle habits such as caffeine intake, smoking, and alcohol consumption. The results showed that there were no significant differences in the treatment outcomes between patients with different lifestyle habits.

### 3.5. Safety assessment

No newly identified adverse events or significant adverse reactions were reported during the study period and the 4-week follow-up period after completion of medication. Only 1 patient had colds for 3 days and recovered.

## 4. Discussion

This retrospective observational case series collected medical records of 35 patients diagnosed with GERD who received outpatient treatment at 18 KM clinics. The participants received HM treatment for 30 days. The baseline characteristics of the patients showed a predominance of female participants, with an average age of 47 years. The HM commonly included herbs such as Pinelliae Tuber, Zingiberis Rhizoma Recens, Poria Sclerotium, Citri Unshius Pericarpium, Cyperi Rhizoma. The study demonstrated significant improvements in GERD symptoms, as indicated by decreases in NRS, GerdQ, and FSSG scores over time. QOL, assessed by EQ-5D-5L and EQ-VAS scores, also showed significant improvements.

Our analysis revealed that patients experienced a reduction in GERD symptoms, as indicated by improvements in NRS, GerdQ, and FSSG scores, following HM. The high medication adherence rate observed in this study (95.9%) suggests that patients were compliant with the prescribed HM. This finding strengthens the validity of the study’s results. Interestingly, although the herbal prescriptions were based on patterns, the most frequently used herbs were consistent in targeting GERD symptoms, irrespective of the specific pattern. Pinelliae Tuber, Zingiberis Rhizoma Recens, Poria Sclerotium, and Citri Unshius Pericarpium were the most frequently used herbs for patients with GERD, and they were commonly added to the basic formulae (water extract) for GERD. Notably, these 4 herbs are key components of *Yijin-tang*, a traditional herbal formula that has been used for centuries to address GERD and has demonstrated effectiveness in various animal models of GERD.^[19,20]^ A previous study conducted in China also reported superior therapeutic outcomes for this herbal formula compared to omeprazole magnesium enteric-coated tablets in patients with GERD.^[21]^ These findings align precisely with a previous review of *Yijin-tang* for GERD based on classical herbal literature.^[22]^ Also, Kuo et al^[23]^ conducted a network analysis of prescriptions in the “Internal Injury” section of the Principles and Practice of Eastern Medicine. They found that the most frequently used herbs in prescriptions for internal injury were Glycyrrhizae Radix et Rhizoma, Atractylodis Macrocephalae Rhizoma, Citri Reticulatae Pericarpium, Panax Ginseng Radix, and Pinelliae Rhizoma. The most common herb combinations were Citri Reticulatae Pericarpium, Glycyrrhizae Radix et Rhizoma, Atractylodis Macrocephalae Rhizoma, and Panax Ginseng Radix, in that order. The centrality analysis indicated that Glycyrrhizae Radix et Rhizoma, Atractylodis Macrocephalae Rhizoma, Citri Reticulatae Pericarpium, and Panax Ginseng Radix were the most central herbs. They primarily included herbs from the *Sagoonja-tang* and *Yijin-tang* families. The resemblance between the network analysis of frequently used herbs for GERD and internal injury prescriptions is noteworthy, suggesting a potential similarity between herbs used for digestive disorders and internal injuries.^[24–27]^

It is noteworthy that the list of frequently used herbs in our study bears similarities to a previous randomized controlled trial (RCT) conducted in Japan, which investigated *Yukgunjatang* for GERD.^[28]^ Additionally, the formula *Naesohwajung-tang*, which combines *Daehwajung-eum* and *Naeso-san*, is a commonly used HM for gastrointestinal symptoms.^[29]^ It includes Pinelliae Tuber, Zingiberis Rhizoma Recens, Poria Sclerotium, Citri Unshius Pericarpium, Cyperi Rhizoma, Coptidis Rhizoma, Massa Medicata Fermentata, Atractylodis Rhizoma, Glycyrrhizae Radix et Rhizoma and Magnoliae Cortex, all of which were included in our list of the top 10 frequently used herbs. A previous RCT confirmed the effectiveness and safety of *Naesohwajung-tang* for digestive symptoms.^[30]^ Considering this research and clinical experience, these 4 herbs-Pinelliae Tuber, Zingiberis Rhizoma Recens, Poria Sclerotium, and Citri Unshius Pericarpium can be recommended when selecting herbs for patients with GERD.

Due to the small sample size of this observational case series, there were some limitations for comparison between each HM. However, we attained the lists of frequently prescribed HM, *Gaewool-Whadam-Jian, Jeungmiyijin-tang, Yukul-tang Gamibang*, and *Yangsayukgunja-tang* from 18 primary KM clinics.

*Hyangsayukgunja-tang* was the most representative formula, which consists of Aucklandiae Radix and Amomi Fructus which are added to *Yuggoonja-tang* (a combination of *Sagoonja-tang* and *Ijin-tang*). *Hyangsayukgunja-tang* showed increased mRNA expression levels of ghrelin and GOAT in rats,^[31]^ and mildly, but significantly, lowered the severity score of GERD in a RCT.^[32]^ *Gaewool-Whadam-Jian* used for GERD, was found to show positive effects in rat experiments.^[33]^ The administration of *Gaewool-Whadam-Jian* improved the transport capacity of the small intestine, suggesting its potential to enhance gastrointestinal function, and it was observed that the intake of *Gaewool-Whadam-Jian* did not cause significant damage to kidney and liver tissues, indicating its relative safety profile. Furthermore, an increase in serum glucose content was noted, indicating a potential impact on glucose metabolism. These findings provide valuable insights into the beneficial effects of *Gaewool-Whadam-Jian* in the context of GERD treatment. *Jeungmiyijin-tang*, first historically included in “Uibangjimnyak” during the Ming Dynasty, was studied for its ability to inhibit pro-inflammatory cytokines (TNF-α, IL-1β, MMP-9) and apoptosis in the esophageal mucosa. The study’s findings suggested that this mechanism offers protection against reflux-induced mucosal damage.^[20]^ In addition, another case study showed that *Jeungmiyijin-tang* plus *Soojeom-san* almost resolved a patient’s GERD symptoms.^[34]^ *Yukul-tang*, in a rat experiment, demonstrated a significant reduction in the incidence of gastric ulcers compared to the control group.^[35]^ A RCT of *Yukul-tang* showed that the observation group had a significantly higher treatment effectiveness rate (100.00%) compared to the control group (91.11%, *P* < .05).^[36]^ *Yukul-tang* effectively improved reflux esophagitis symptoms, resulting in patient recovery with minimal adverse reactions, supporting its safety and reliability.

The analysis of GERD scores, including NRS, GerdQ, FSSG, EQ-5D, and EQ-VAS, demonstrated significant improvements over the 60-day treatment period. The decrease in NRS scores indicates a reduction in GERD symptom severity. Similar trends were observed in the GerdQ and FSSG scores, further confirming the effectiveness of HM in alleviating symptoms. In a previous study among patients with GERD conducted in South Korea,^[10]^ the average NRS score was 5.8 ± 2.2, which was similar to the results of our study. The improvements in EQ-5D and EQ-VAS scores suggest enhanced overall well-being and improved health-related QOL.^[37]^ According to a systematic review of HM for GERD, the total score of symptoms, FSSG scores, GerdQ score showed no statistically significant difference.^[38]^ Subgroup comparison analysis revealed that treatment effectiveness did not differ significantly based on lifestyle habits such as caffeine intake, smoking, and alcohol consumption. This suggests that HM may be effective across different patient subgroups, regardless of these lifestyle factors. However, larger studies with more diverse populations are necessary to further explore this finding.

There have been few studies which assessed the effectiveness of HM within a 30-day period, which may be due to time and cost constraints. The strengths of this study are that our results showed noticeable improvements in the diverse GERD scores after HM, and that we used this detailed questionnaire at real world primary clinics. When planning the treatment strategy for patients with GERD, it is important to know which treatment is effective for the management of symptoms. The current study showed that the HM was particularly effective in the improvement of all functions and delayed recall, all the scores of NRS, GerdQ and FSSG. As there were no serious adverse events, HM seems to be safe based on our limited cases.

There are some limitations to our study. First, it is an observational case series without a control group, and the effect size of HM for GERD in our study has the potential to be overestimated. Second, due to the small sample size, it was not sufficient to compare the difference in the effects of pattern formula. Third, practitioners were free to choose herbal formula in our study but did not collect data about the detailed reasons for the prescription. Further studies are required to evaluate the effectiveness of each personalized HM based on patterns. Despite these limitations, this study is notable in that it is the first study to investigate diverse herb formulae for GERD in real world practice in 18 primary KM clinics.

## 5. Conclusion

The findings of this study support the effectiveness and safety of HM in reducing GERD symptoms and improving the QOL of patients in primary KM clinics. The high adherence rate to medication indicates patient compliance, enhancing the reliability of the results. The most frequently used herbs, such as Pinelliae Tuber, Zingiberis Rhizoma Recens, Poria Sclerotium, and Citri Unshius Pericarpium may play significant roles in GERD symptom management. However, the small sample size and short duration of the study highlight the need for larger and longer-term studies. Nevertheless, this study contributes to the growing body of evidence supporting HM as a potential alternative treatment for GERD.

## Acknowledgments

We extend our sincere thanks to the Association of Korean Medicine for their contributions to make this research possible.

## Author contributions

**Conceptualization:** Sungha Kim.

**Data curation:** Ki-Byoung Kim KMD.

**Formal analysis:** Ojin Kwon.

**Funding acquisition:** Changsub Yang KMD.

**Investigation:** Wongu Lee KMD, Ki-Byoung Kim KMD.

**Methodology:** Ojin Kwon, Ki-Byoung Kim KMD.

**Supervision:** Sungha Kim.Validation: Ki-Byoung Kim KMD.

**Writing – original draft:** Hesol Lee KMD.

**Writing – review & editing:** Wongu Lee KMD, Ki-Byoung Kim KMD, Sungha Kim.
